# Catastrophic factors involved in road accidents: Underlying causes and descriptive analysis

**DOI:** 10.1371/journal.pone.0223473

**Published:** 2019-10-09

**Authors:** Imran Ashraf, Soojung Hur, Muhammad Shafiq, Yongwan Park

**Affiliations:** Department of Information & Communication Engineering, Yeungnam University, Gyeongbuk, Gyeongsan-si 38541, Republic of Korea; University of British Columbia, CANADA

## Abstract

South Korea is ranked as 4th among 34 nations of the Organization for Economic Cooperation and Development with 102 deaths in road accidents per one million population. This paper aims to investigate the factors associated with road accidents in South Korea. The rainfall data of the Korea Meteorological Administration and road accidents data of Traffic Accident Analysis System of Korea Road Traffic Authority is analyzed for this purpose. In this connection, multivariate regression analysis and ratio analysis with the descriptive analysis are performed to uncover the catastrophic factors involved. In turn, the results reveal that traffic volume is the leading factor in road accidents. The limited road extension of 1.47% compared to the 4.14% per annum growth of the vehicles is resulting in road accidents at such a large scale. The increasing proportion of passenger cars accelerate road accidents as well. 56% of accidents occur by the infringement of safety driving violations. The drivers with higher driving experience tend to have a higher accident ratio. The collected data is analyzed in terms of gender, driver experience, type of violations and accidents as well as the associated time of the accidents when they happen. The results indicate that 36.29% and 53.01% of accidents happen by male drivers in the day and night time, respectively. 29.15% of crashes happen due to safety infringement and violations of 41 to 60 years old drivers. The results demonstrate that population density is associated with the accidents frequency and lower density results in an increased number of accidents. The necessity of the state-of-the-art regulations to govern the urban road traffic is beyond dispute, and it becomes even more crucial for citizens’ relief since in our daily lives road accidents are getting more diverse.

## Introduction

In a modern era, it is undeniable that the transportation system improves humans living standard as well as it contributes to the economic growth of a country. It makes people moving themselves and their goods more easily to fulfill their tasks across the world. It is inevitably considered as a symbol of social well-being and self-image of a nation [1]. The terms like the transportation industry’s Gross Domestic Product (GDP), Transportation-related GDP and transportation made GDP are often referred to measure the share of transportation in a country’s economy [2]. With the increase in population and the growing competition to provide luxurious traveling services, transportation infrastructure is becoming larger and complicated day by day. The transportation system is, therefore, the most essential element of human civilization and it could become the most harmful element if not properly managed otherwise.

In one report, the World Health Organization (WHO) articulates that the proportion of road accidents fatalities to total deaths in the world has grown by 2.2% from 1.255 million deaths in 2012 [3]. The accidents rate was increased by 0.3% from 2000 to 2012 despite the efforts served in terms of better road and laws enforcement. This is how WHO ranks road accidents as the 9th leading cause of deaths with 17.7 per 100,000 population in the world, which is sadly very close to that of dangerous diseases like diabetes, diarrhea, HIV/AIDS, etc. In addition, approximately 30 to 50 million population are either injured or permanently disabled every year. Moreover, road accidents every year cause great financial havoc of $518 billion and so costing countries 1% to 2% of their individual GDP alone.

**Fig 1:**
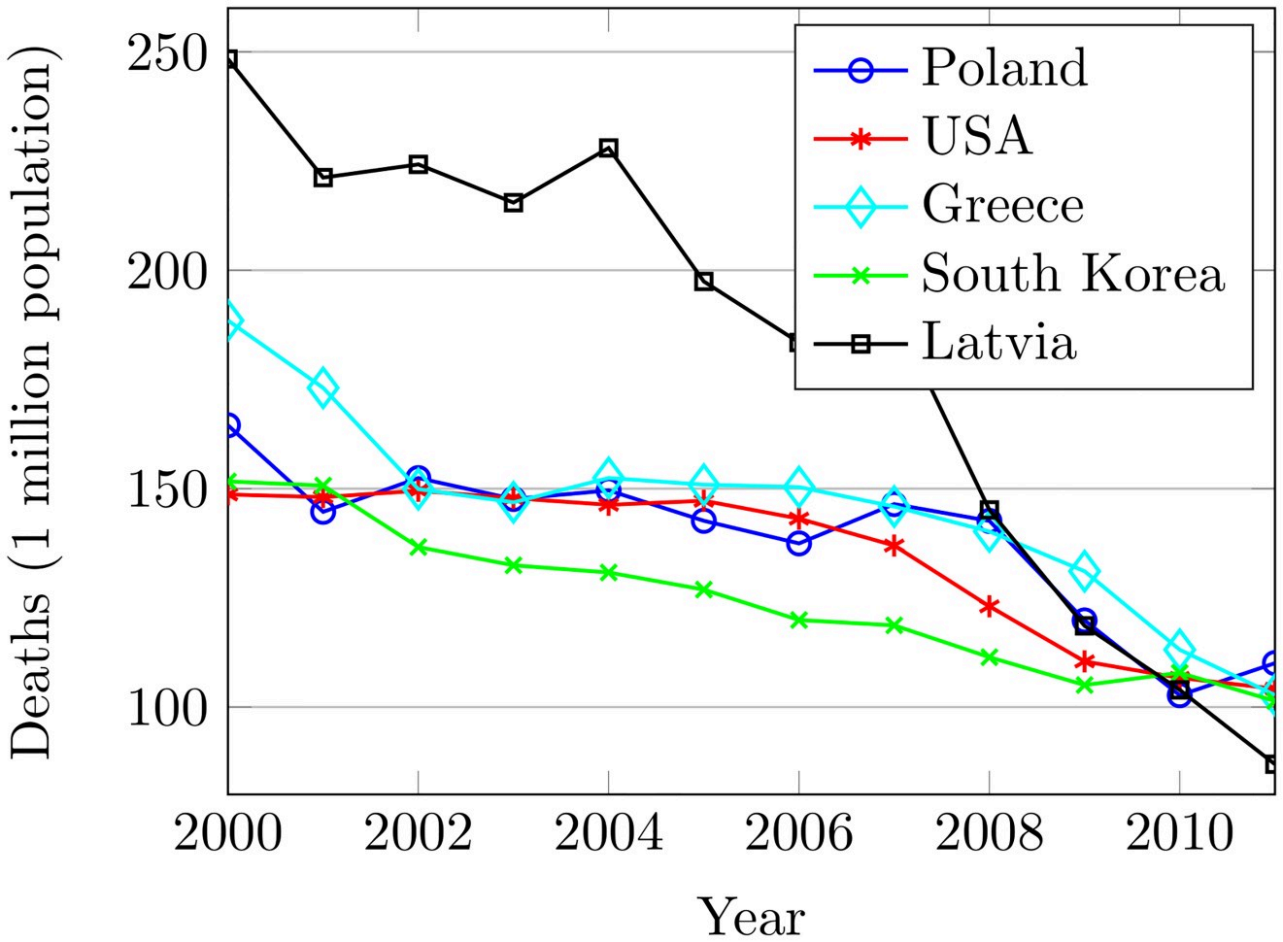
OECD countries with the highest deaths per one million population from 2000 to 2011.

The Organization for Economic Cooperation and Development (OECD) is an alliance of 34 member governments including South Korea. The member countries in OECD also suffer from road accidents. Fig 1 shows statistics of five OECD countries having the highest deaths in accidents for 2000 to 2011. Worse still, South Korea is one of those countries. Korea has a total of 104,236 km road length comprising 3,447 km as national expressway, 13,905 km as national highway and 86,884 km as provincial roads [4]. There were 18,870,533 registered vehicles in 2012 and so every third person has his/her own vehicle in South Korea. Despite the virtues, the transportation system in Korea is being crowded and getting complex every day and so leads to increasing the disastrous crashes.

**Fig 2:**
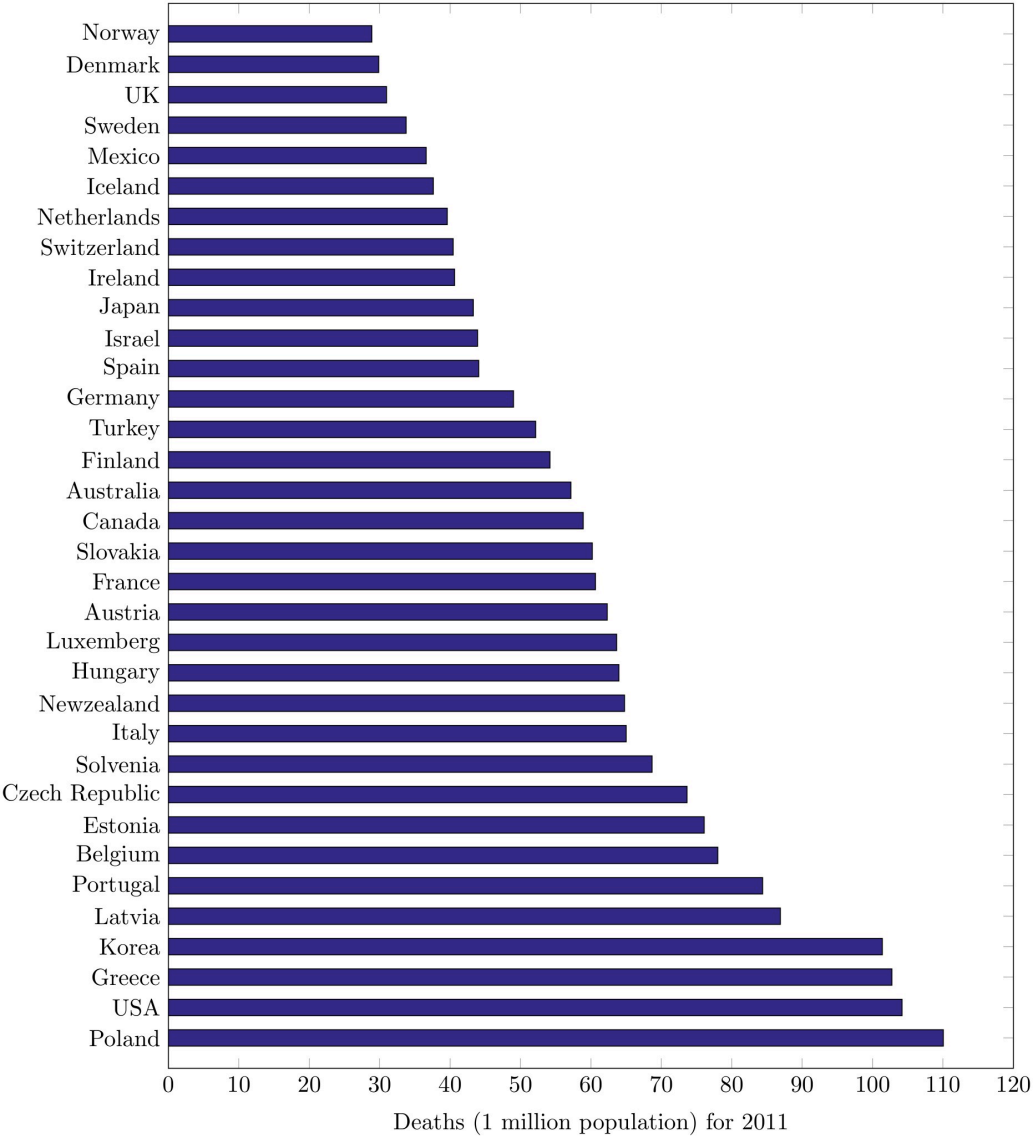
The deaths in accidents for OECD countries in 2011.

Korea has 4th highest accident rate (after Poland, USA, and Greece) in OECD nations with 102 deaths per one million population [5]. Fig 2 shows statistics of 2011 for all the OECD countries. While at the same time many other underdeveloped OECD countries like Austria, Chile, Iceland, and Spain have lower death rates than that of South Korea. So, investigating the paramount causes and effects of road crashes could help in the design of their countermeasures. It will not only save human lives but can certainly reduce the overwhelming effects of financial losses as well. In this paper, the aim is to investigate the research questions: 1) What are the possible causes of urban road crashes in South Korea?, 2) What is the relationship between the causing factors and accidents?, 3) How the effects of such causing factors can be reduced?

The key contributions of this study can be summarized as follows:

- This study considers the data of accidents and that of their associated weather conditions to perform the analysis. For that purpose, the data is gathered from a variety of state-of-the-art sources for more than one decade considering a number of affecting variables.
- An in-depth investigation is conducted regarding the accident types that are caused by the gender of the driver during the day and night time with their driving experience and violations leading to the accidents.
- Single and multivariate regression analysis along with descriptive analysis is carried out to highlight various uncovered aspects like age, gender, experience, violation type, traffic volume, etc. to understand their individual as well as the collective effects on accidents.
- This study suggests propositions to mitigate the risk of accidents for elderly drivers especially during the night-time and bad weather conditions, which are equally helpful to the Governments in the design of traffic laws to govern the urban roads traffic.

The rest of this paper is organized as follows. Section summarizes research works related to current study. The collected data and their sources are elaborated in Section. Section presents the methodology of the analysis. Results and discussions are described in Section. The key results and insights along with the propositions of the study are presented in Section. Finally, the study is concluded in the last section.

## Related work

Road accidents have been an active research topic of the academia and industry for the last few years. It is mainly focused because of the enabling demand of safety conditions and automation in vehicles. In the existing literature, we can find several research papers as [6–9] that have focused the causal relationship among road conditions, driver conditions such as drunken driving, driver behavior, age, gender, etc., weather conditions like precipitation, rain, snow, fog, etc., and road accidents rates in the different locations. Such research is based on the application of various techniques, like Bayesian model [10] Poisson model [11], and regression analysis [12]. [13] explores the relationship between traffic congestion and road accidents for M25 London orbital motorway. This study aims to articulate that there exists an inverse relationship between traffic congestion and road accidents. Spatial and non-spatial Poisson models are used to show that traffic congestion has very little or even no impact on the number of road accidents. The investigation does not consider the influence of other motorways and major roads connected to M25 motorway. Such conclusions may be non-deterministic without examining the impact of traffic from attached small roads and cross junctions.

On the other hand, [14] investigates the connection between precipitation and the number of crashes on roads from 1975 to 2000 and show a negative relationship between precipitation and severe crashes in the United States per month. This research concludes that the risk of a road crash is increased with increase in the time since the last precipitation. However, it is generally believed that rain can directly affect the frequency of crashes on roads. For instance, [15] evaluates the influence of rain on road crashes for the period of 1987 to 2002 in Melbourne. Therein, the data is split into three segments to enable match-pair analysis approach to find out an association between the rain and the increased volume of accidents. This study validates that rain is a road traffic hazard, and so affect the number of accidents.

In [16], authors investigate the impact of traffic volume and lighting conditions on road accidents. The traffic volume measured prior to the occurrence of the accident has proved a strong relationship between the driving speed and traffic collisions. It also analyzes that wet roads play in hit-object and multiple vehicle crashes. We can also find out that the severity of accidents has a close association with high traffic volume than the driving speed. [17] analyzed the weather’s influence on the traffic volume in Melbourne from 1989 to 1996. This work found a strong correlation between rain and traffic volume reduction during the day and night time of the spring and winter. This study concludes that the rain of 2mm to 10mm range causes 2% to 3% reduction in the traffic while 2mm to 5mm rain in spring leads to a 3.43% decrease in the traffic volume. Therein, the separate consideration of the day and night time results in different findings of 1.86% reduction in the winter and 2.16% reduction during the spring day. Similarly, the nighttime reduction is about 0.87% in winter and 2.91% in spring nights. [18] carry out the study on Tokyo Metropolitan Expressway to find the impact of rain on the change in traffic volume and traffic accidents for the period of 1998 to 2004. A total of 42,041 accidents for no rain hours and 5,700 accidents for rainy hours are observed. This study shows that an average rate of 0.85 accidents/hour in no rain while 1.5 accidents/hour in rain are found in those six years of the period. Besides rain, other road and traffic factors may result in a variety of collision types and cause different accident frequency and severity [19].

In [20], authors find out the significance of specific road segments on road accidents for a Belgian periurban area. This study reveals that accidents happening in the “black” zones result from left-turns, pedestrian hits, vehicle run-off-roadway, and rain. Culprits for accidents outside the “black” zones are left turns at intersections with traffic signals, head collisions, and drunken driving. [21] investigates the relationship between road accidents and driver’s gender by performing a statistical analysis on the variables including distance traveled, environmental factors, driver’s age, and gender from low, middle, and high-income regions. This study further emphasizes that a significantly higher accident rate is associated with male drivers in normal driving conditions. However, in changed weather conditions accident rate variations are sufficiently reduced between the male and female drivers. [22] considers the effect of change in speed near the Automated Speed Enforcement System (ASES) on road accidents in South Korea. ASES is deployed to control the speed of vehicles in specialized areas like schools, parks, industry, and so on. Therein, the alarms in the vehicles through voice messages or signboards of approaching ASES zones are focused in order to avoid traffic accidents. This study shows that locations near ASES checkpoints have a 7.6% drop in crash rate. On the contrary, ASES is responsible for an average increase of 11% crashes due to sudden speed reduction while approaching ASES implemented areas.

In [23], authors analyzed old driver’s behavior in road accidents and show that 97% of the crashes are the outcome of judgmental errors by the drivers aging 70 years or above. More prevalent errors that this study points out are inadequate surveillance, misjudgment of the distance between vehicles, medical events and days dreaming. The gap between vehicles or other vehicle’s speed miscalculation and inappropriate surveillance are most commonly found among the elderly female drivers. [24] finds out the influence of vehicle’s speed on the number and severity of injuries. The power model for speed and road accidents show that a strong statistical relationship exists between the vehicle speed and road accidents. This study further points out that the relationship may not be necessarily linear, but chances of road crash increase exponentially with the increase in speed.

In [25], authors developed unbalanced panel data and used hourly records to analyze the likelihood of hourly crashes on highway influenced by real-time weather. Results show that weekend indicator, November indicator, low-speed limit and long remaining service life of rutting indicator are found to increase the crash likelihood. The study confirms that real-time weather, road surface, and traffic conditions have a significant impact on road crashes. In the same way, authors in [26] performed an analysis of new vehicles e.g., powered two-wheelers to understand their behavior which causes a discrepancy in road traffic.

The findings of the aforementioned studies nevertheless cannot be directly applied to South Korea due to a variety of reasons. The data gathering process may not be transparent, complete or truthful. Since the data are mostly gathered by police stations or insurance agencies. So, the statistics may be exaggerated on the part of both victims and offenders [27, 28]. Furthermore, accidents are not reported on many occasions when the involved parties reach on an agreement. The analysis is performed with respect to a particular area, like a city or country with different demographic variables. In the same fashion, accidents may also depend on the infrastructure, economy, culture, social norms and gender discrimination prevailing in a subject area. So, an analysis cannot hold true for different areas. This is how a separate statistical analysis is desirable by considering the environmental, weather and driving conditions for South Korea so as to better understand the associated factors of road accidents. This study presents a separate multivariate regression analysis to explore the leading causes of road crashes in order to find out the suggestions that could help in the design of transportation policies.

## Data collection

**Fig 3:**
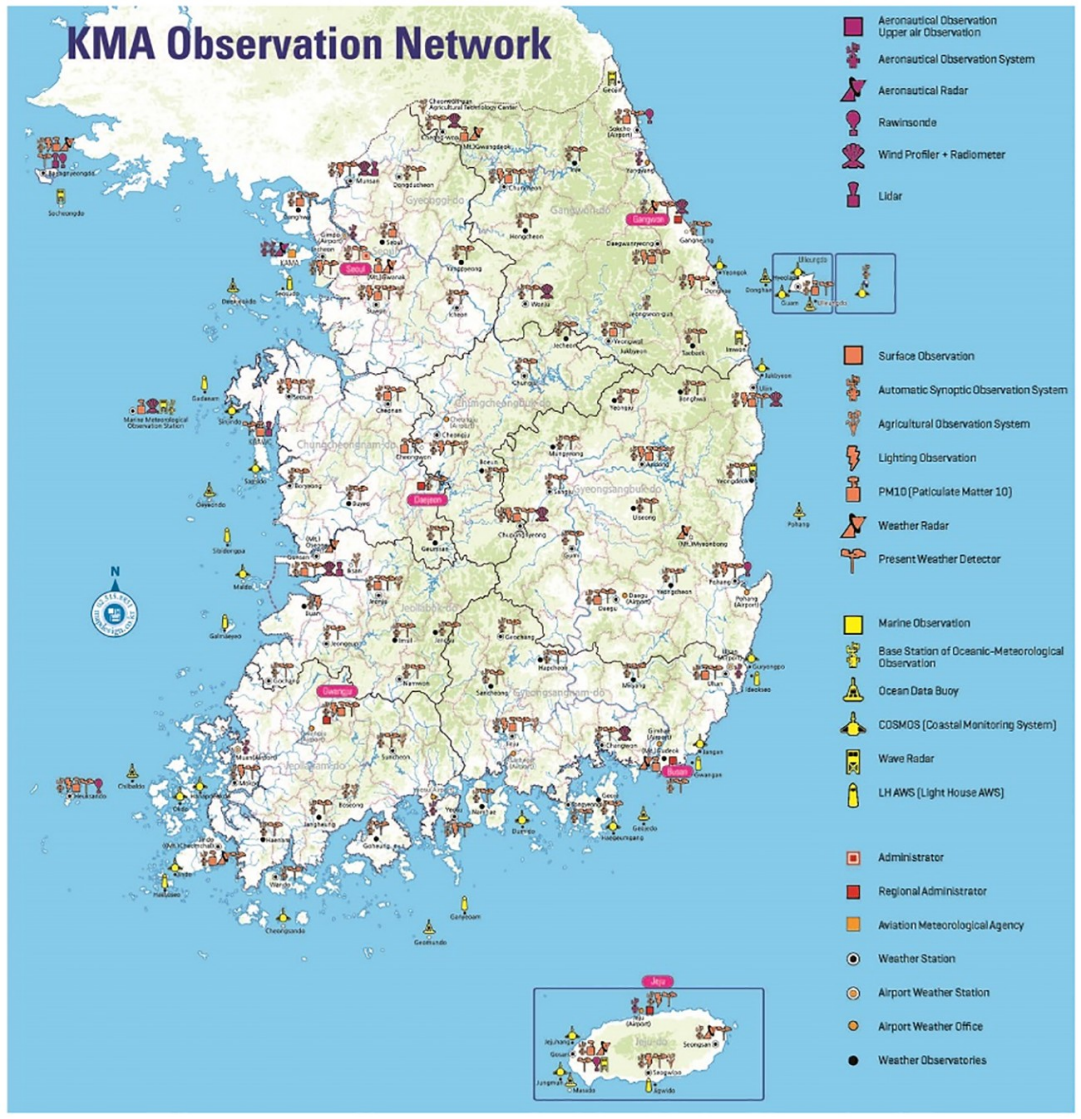
The KMA observation network in 2011.

For the analysis purpose, the weather data is collected by the Korea Meteorological Administration (KMA) from 2000 to 2012. KMA is the national weather data collection and forecasting center of South Korea. KMA formally started weather observation by radar in 1963. Fig 3 shows the prospect of the KMA observation network [29]. It has a widely scattered observatory network of radar and satellite stations throughout the country. This network has 94 weather stations, 464 Automatic Weather Stations (AWS), and 10 upper-air observation stations that include weather radar observations and aviation meteorological stations. The road accidents statistics are from 2000 to 2012, which are collected by the Traffic Accident Analysis System (TAAS) of Korea Road Traffic Authority (KoROAD). TAAS gathers information from police stations, insurance companies, and mutual aid associations. It makes the accident data available to the public every year. In addition, data regarding driving conditions, road safety, and health statistics are collected from the International Road Traffic and Accident Database (IRTAD) [30], OECD and the World Health Organization (WHO) to establish our analysis.

## Methodology

This study conducts the regression analysis to identify the relationships between accidents and various factors like weather, road conditions, driving behavior, etc. The regression analysis is a widely used technique to investigate the interrelationship among a group of variables. It first analyzes the functional relationship among variables and then checks the accuracy of the relationship. In this study, the single linear regression and the multivariate linear regression analysis with the descriptive analysis are performed to investigate the effect of individual factors. The simple linear regression is helpful in understanding the driving factors that could affect the output (or target) individually [31]. However, considering the multiple variables at the same time often lead to the most meaningful knowledge from the data. For example, studying the impact of driving experience on accidents maybe a piece of simple information. When such a driving experience with other factors including driving environment (i.e., day/night) and gender is considered, it makes better sense to highlight the root causes otherwise. Multivariate regression is performed to draw conclusions based on the collective influence of various factors. The regression model is given as,

$$\begin{array}{c} y=const+\beta_{1}x_{1}+\beta_{2}x_{2}+,\ldots ,+\beta_{n}x_{n}+\mu , \end{array}$$ (1)

where *y* is a dependent variable, *const* is an intercept, *β* values represent coefficients for regressors *x* and *μ* denotes error constant to show the discrepancy or failure of the data model. The dependent variable *y* accounts for the frequency of accidents determined by *x* variables including rain, the gender of the driver, traffic volume, road length, etc. However, *μ* represents a portion of the accidents inexplicable by these factors.

The *ρ* − *value*, and *R*^2^ is used to evaluate the performance of the regression model and interpret the results. The *ρ* − *value* is applied to test the hypothesis in a model. It is also called the marginal significance level [32]. It tests the null hypothesis *H*_*o*_, which means that there is no significant relationship between the predictions and regressors. A low *ρ* − *value* suggests that the prediction is meaningful and that the null hypothesis can be rejected. *R*^2^ is called the *goodness-of-fit* index, which is calculated in [31] as follows,

$$\begin{array}{c} R^{2}=1-\frac{SSE}{SST} \end{array}$$ (2)

where,

$$\begin{array}{c} SSE=\sum {\left(y_{i}-\hat{y_{i}}\right)}^{2}, \end{array}$$ (3)

and,

$$\begin{array}{c} SST=\sum {\left(y_{i}-\bar{y_{i}}\right)}^{2}. \end{array}$$ (4)

where *SSE* stands for the sum of squared residuals or errors while *SST* represents a total sum of squared deviations of *y* from its mean value. The value of *R*^2^ varies between 0 and 1 [33]. If *R*^2^ is close to 1 it implies that the variables are perfectly related with perfect goodness of fit. Conversely, if *R*^2^ approaches 0, it then infers that there is no relationship between the given variables and the regressors do not explain the prediction.

## Analysis and results

**Fig 4:**
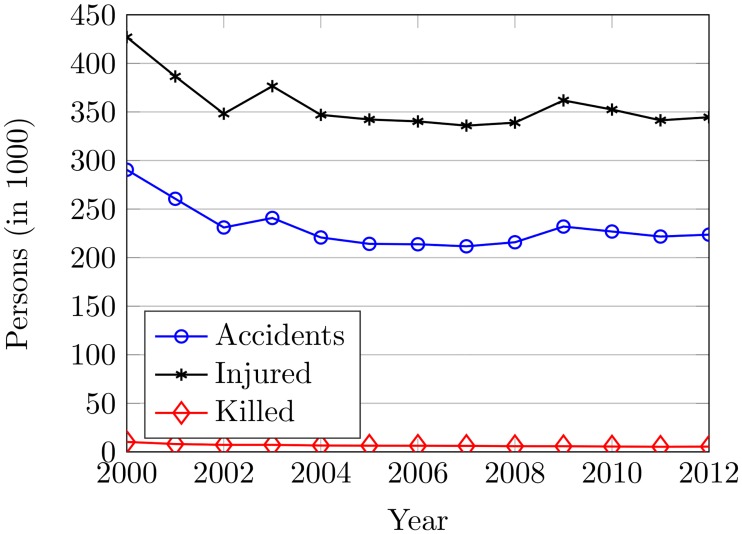
The road accidents in South Korea from 2000 to 2012.

In the analysis, various factors are considered including rain, driver’s age/gender, driving experience, road type, vehicle type involved in accidents, alcohol intoxication, violation type causing the accident, traffic volume, and lighting conditions so as to find their individual impact on the accidents. The accident trends from 2000 to 2012 are shown in Fig 4, which exhibits that accidents in South Korea have been reduced since 2000. However, the frequency of accidents is found higher because it is not in accordance with the economic and demographic conditions of Korea. From 2000 to 2002 and from 2003 to 2006, accidents are potentially decreased. Nevertheless, no decrease in accidents is noticed from 2007 and onward. From 2011, accidents are increased by 0.08% despite the government policies and infrastructural improvements. The accidents are decreased by 20% in 2002 compared to 2000 and 2011 while the decrease in the number of accidents was only 7.13% since 2003.

### Factor analysis

In this section, different factors which give rise to the number of accidents are discussed through the linear regression, descriptive and ratio analysis. Further, an investigation of the causes and effects of the associated factors involved in accidents is proffered.

#### Effects of rain on accidents

**Fig 5:**
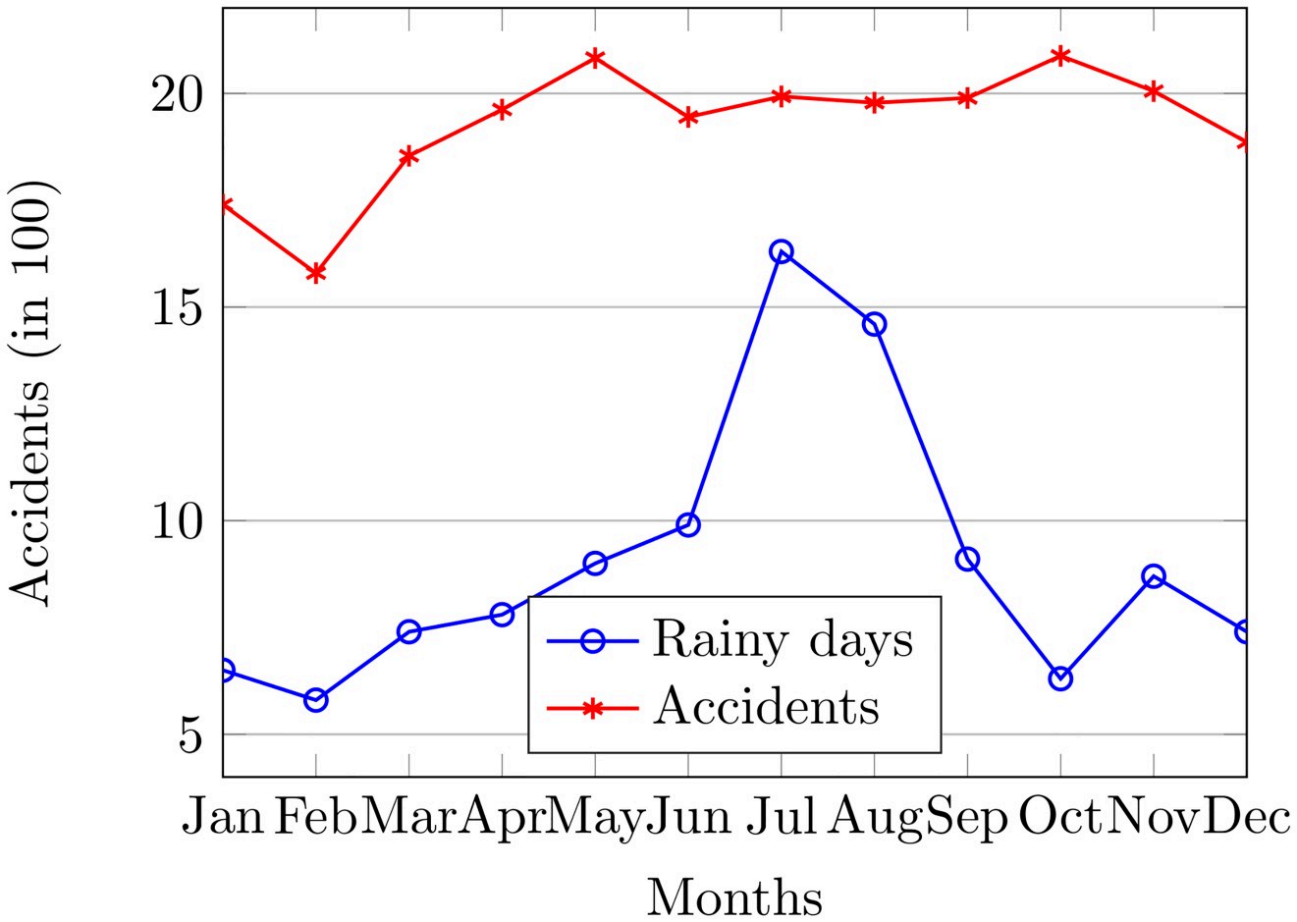
The rainy days vs. accidents in South Korea from 2000 to 2012.

The influence of rain on road accidents is investigated by considering average rainy days and average accidents for each month from 2000 to 2012 as shown in Fig 5. Therein, the purpose of the average calculation is to get the normalized value for rainy days and accidents. It shows the average rainy days and average accidents. For this purpose, the rain quantity can simply be calculated as,

$$\begin{array}{c} r_{q}=t\times p \end{array}$$ (5)

where *r*_*q*_ shows the rain quantity in mm per hour, *t* account for time in hours and *p* represents precipitation rate. The rainy hour *r*_*h*_ is defined as follows,

$$\begin{array}{c} r_{h}=\{\begin{array}{cc} r_{q}>5.05\text{mm} & 1\\ otherwise & 0 \end{array} \end{array}$$ (6)

Please note that the value of *r*_*q*_ is defined with respect to *light rain*, and *moderate rain* whose individually quantities are 2.5 mm and 7.6 mm per hour, respectively [34]. The light rain can slightly impact road accidents compared to moderate and heavy rains. So, the average value of light rain and moderate rain for a rainy hour is considered.

Fig 5 shows that the slopes of accidents and rainy days from January to May, November, and December are almost symmetrical. However, it does not show any connection for the rest of the months which implies that rain is not the sole contributor to road accidents in South Korea. The accident rates from 2000 to 2012 and their average is calculated for each month to corroborate the facts.

The calculations are performed for the number of rainy days of each month from 2000 to 2012. The aim of regression analysis is twofold: 1) to investigate whether the rain has any influence on the frequency of accidents, 2) to check if the high rain has any association with the number of accidents. Table 1 shows the results of regression analysis at 95% confidence level. This could be helpful to notice that the *R*^2^ value given in Table 1 indicates that only 11% of the accidents can be caused by rain with 0.3148 P-value which is significant.

**Table 1:** The rainy days and accidents on average for each month from 2000 to 2012.

Months	Rainy days	No. of accidents	Accidents for rainy days
January	6.5	17398	3647
February	5.8	15793	3271
March	7.4	18541	4425
April	7.8	19622	5101
May	9.0	20827	6046
June	9.9	19449	6418
July	16.3	19929	10478
August	14.6	19782	9316
September	9.1	19893	6034
October	6.3	20879	4243
November	8.7	20054	5815
December	7.4	18858	4501
*R*^2^	0.112	P-value	0.314

#### Role of gender in accidents

The study of male and female drivers has been the core of various research works. Therein research findings have established the concept that male and female driving has altogether different characteristics. Male drivers have a significantly higher accident rate than that of the female counterparts irrespective of their age [21, 35, 36]. However, this study performs the calculations to analyze the effect of gender on road accidents from 2009 to 2012. Table 2 shows the accident ratios, which indicates that the male drivers have higher accident rates as a higher number of accidents per male driver have occurred for the analysis period. “Acc/1000 drivers” column shows that this ratio is approximately three times higher than that of female drivers’. The accident rate for female drivers is pretty low compared to male drivers. According to the licensed female driver ratio, the number of female drivers is increasing than that of male drivers as 38.75, 39.12, 39.55 and 40.09 for 2009 to 2012, respectively. The ratio of male-to-female accidents has observed an increase in that period. The lower accident rates for female drivers can be attributed to several reasons. Among the most persuasive are: lower speed driving, driving on less dangerous roads, smaller distance traveled and careful driving. The results of regression analysis at 95% confidence level for male and female drivers from 2009 to 2012 are exhibited in Table 2, which shows that the increase in accidents is attributed to 74% increase in the number of male drivers while 22% with the female drivers with significant and insignificant P-values for male and female drivers, respectively. The findings are in accordance with the established fact that male drivers are more exposed to road accidents than female drivers. It is observed that the accident rate of the male driver is 3.17 times higher on average than that of female drivers, which is a significant difference.

**Table 2:** The accidents rate by male and female drivers per annum from 2009 to 2012.

Year	Male		Female		* Ratio
	Accidents	Acc/1000 drivers	Accidents	Acc/1000 drivers	
2009	187,662	11.86	37,462	3.74	3.17
2010	182,882	11.38	37,135	3.60	3.16
2011	177,688	10.79	36,928	3.43	3.15
2012	178,745	10.56	37,208	3.28	3.21
Avg.	-	11.15	-	3.51	3.17
*R*^2^	0.7429	-	-	0.2237	-

* Ratio is for male-to-female accidents.

#### Road traffic volume and accidents

The road traffic volume is of the prime interest among the road factors related to the accidents. Previous researches [13, 37–39] have established a distinctive correlation between traffic volume and road accidents. The influence of traffic volume and congestion on accidents is not very well studied. However, different findings state its direct and inverse relationship on the frequency and severity of accidents [13, 38]. Similarly, research works [40, 41] look into single and multi-vehicle truck accidents caused by traffic volume and wet rods. Research findings indicate that snow road surface is to be modeled as random-parameters in the single and multi-vehicle accidents. The light traffic is found to have a complex interaction among traffic volume and accident-injury severity. Research findings reveal that high traffic volume has a large impact on the frequency of accidents, yet it does not have an intense impact on injury severity. Similarly, traffic flow is found to intensify the injury severity of both drivers caught in rear-end car accidents.

**Fig 6:**
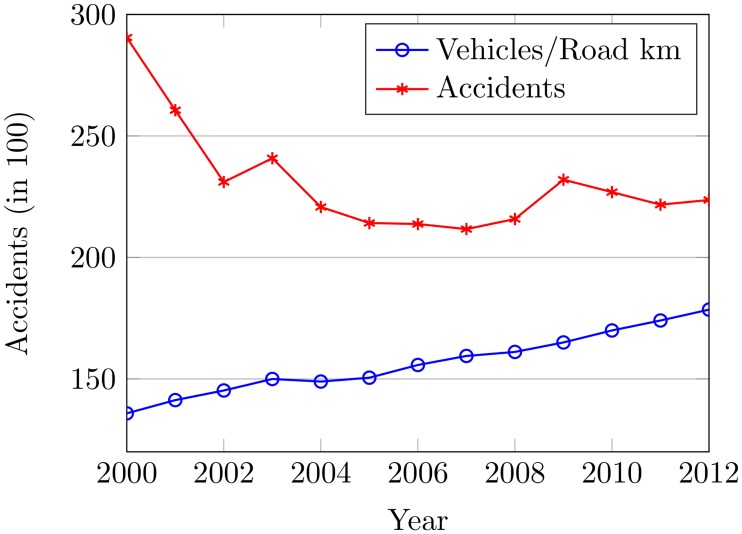
The traffic flow and accidents from 2000 to 2012.

The average values of traffic volume are shown in Fig 6 for individual months compared to the average number of accidents from 2000 to 2012. It can be seen that the number of vehicles per road km compared to the number of accidents has a decreasing trend from 2000 to 2007 in spite of the increase in vehicles and then accidents are increased from 2008 to 2012. Such a decrease in accidents might be attributed to different transport policies like the speed limit, BAC laws, road infrastructural extension, improvement, etc. However, the increase is inexplicable.

**Fig 7:**
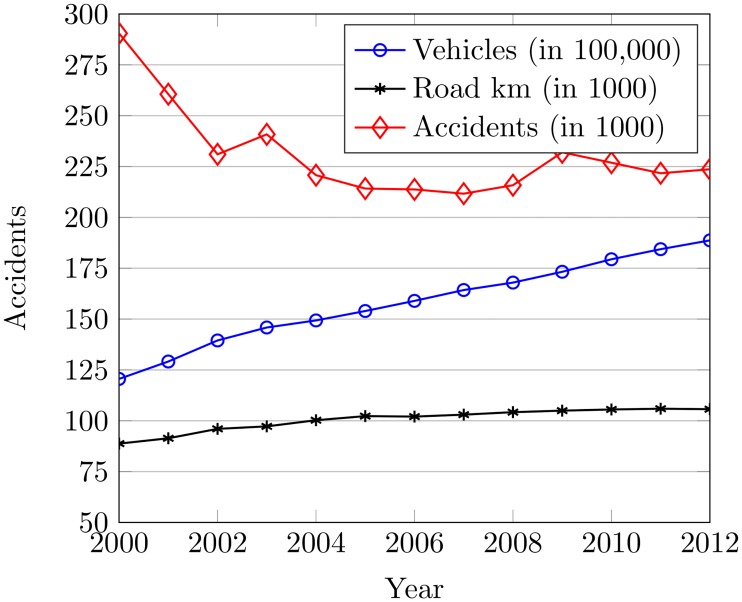
The registered vehicles with road length vs. accidents from 2000 to 2012.

The registered vehicles and road in km are drawn against the number of accidents to analyze the relationship between traffic volume and road accidents. Fig 7 shows a gradual decrease in accidents from 2000 to 2007 even the number of registered vehicles is increased. The number of accidents is decreased as the road length is extended in that period. The road extension from 2000 to 2007 is 88,755 km to 103,019 km, which is measured as 16.04%. Nevertheless, the scenario is changed for 2008 to 2012 where the accident rate is increased with the increase in the number of vehicles. The preeminent cause for such increase is a low incremental change in road length even so vehicles are increasing at an accelerated rate. The road extension from 2007 to 2012 is 1.42% that is noticed from 103,019 km to 105,703 km. This is how an unbridgeable gap between vehicles and road length has ultimately caused high congestion with large traffic volume which leads to an increase in accidents rates.

**Fig 8:**
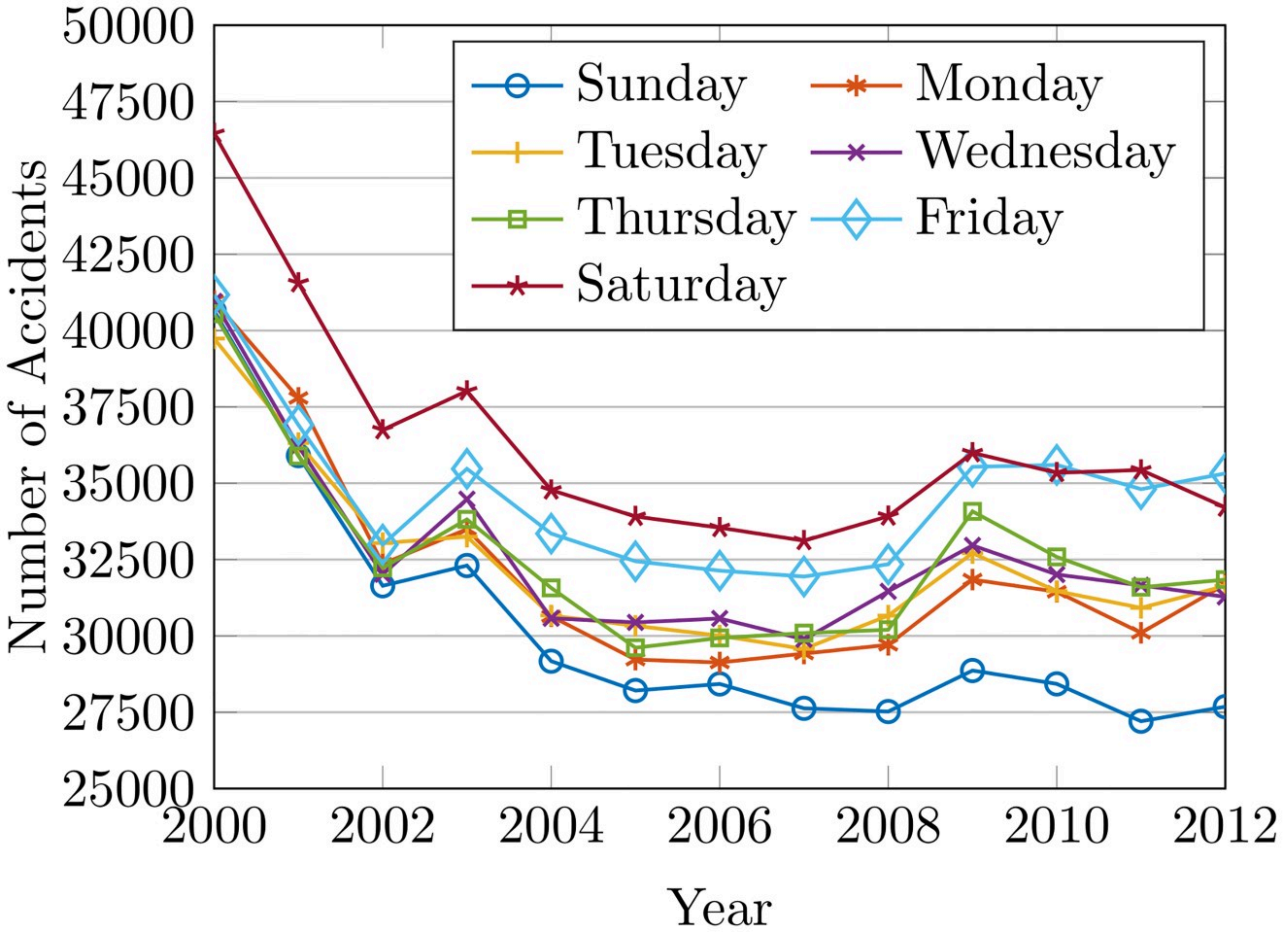
The accidents by days of the week from 2000 to 2012.

The road length and vehicles can cause 85% of the total accidents with significant P-value and very low standard error as shown in Table 3. 4.14% increase in registered vehicles on average can be seen for the period while road extension is at an average rate of 1.47% which causes to elevate traffic volume and congestion and so leading to increase in accidents. The registered vehicles have been increased by 69% while the road extension is only 20% since 2000, which creates a huge gap between the number of vehicles and the available roads. Fig 8 illustrates the number of accidents by days of the week from 2000 to 2012. This is to verify the relationship between traffic volume and the number of accidents. An increase in the accident rate by Friday and Saturday is observed. This is because of high congestion and traffic volume due to weekend travel.

**Table 3:** The vehicles and road vs. accidents from 2000 to 2012.

Year	Reg. veh.(1000)	Road length (km)	Road/10,000 veh.	Change in veh. (%)	*Change in road (%)
2000	12,059	88,775	73.62	8.02	1.42
2001	12,914	91,397	70.77	7.09	2.95
2002	13,949	96,037	68.85	8.02	5.08
2003	14,586	97,253	66.67	4.57	1.27
2004	14,934	100,278	67.15	2.38	3.11
2005	15,396	102,293	66.44	3.10	2.01
2006	15,895	102,061	64.21	3.24	-0.27
2007	16,428	103,019	62.71	3.35	0.94
2008	16,794	104,236	62.07	2.23	1.18
2009	17,325	104,983	60.60	3.16	0.72
2010	17,941	105,565	58.84	3.56	0.55
2011	18,437	105,931	57.45	2.76	0.35
2012	18,870	105,703	56.01	2.35	-0.22
Avg.	15,810	100,579	64.26	4.14	1.47
R^2^	0.855	P-value	0.0004	Ref.	Significant

* Negative (-) sign for ‘change in road’ shows the reduction of the road due to deterioration and natural disaster.

#### Impact of road structure on accidents

Apart from the weather, vehicle speed, driver age, and road type are other substantial factors. These factors are investigated to evaluate their influence on road accidents. Various road parameters such as length of the road section, average speed limit, road lanes, road width, intersections, and pavement type have a prominent impact on accident rates [42–44]. The analysis of accidents regarding road type where the accident occurred could describe the relationship between the type of road and its impact.

**Fig 9:**
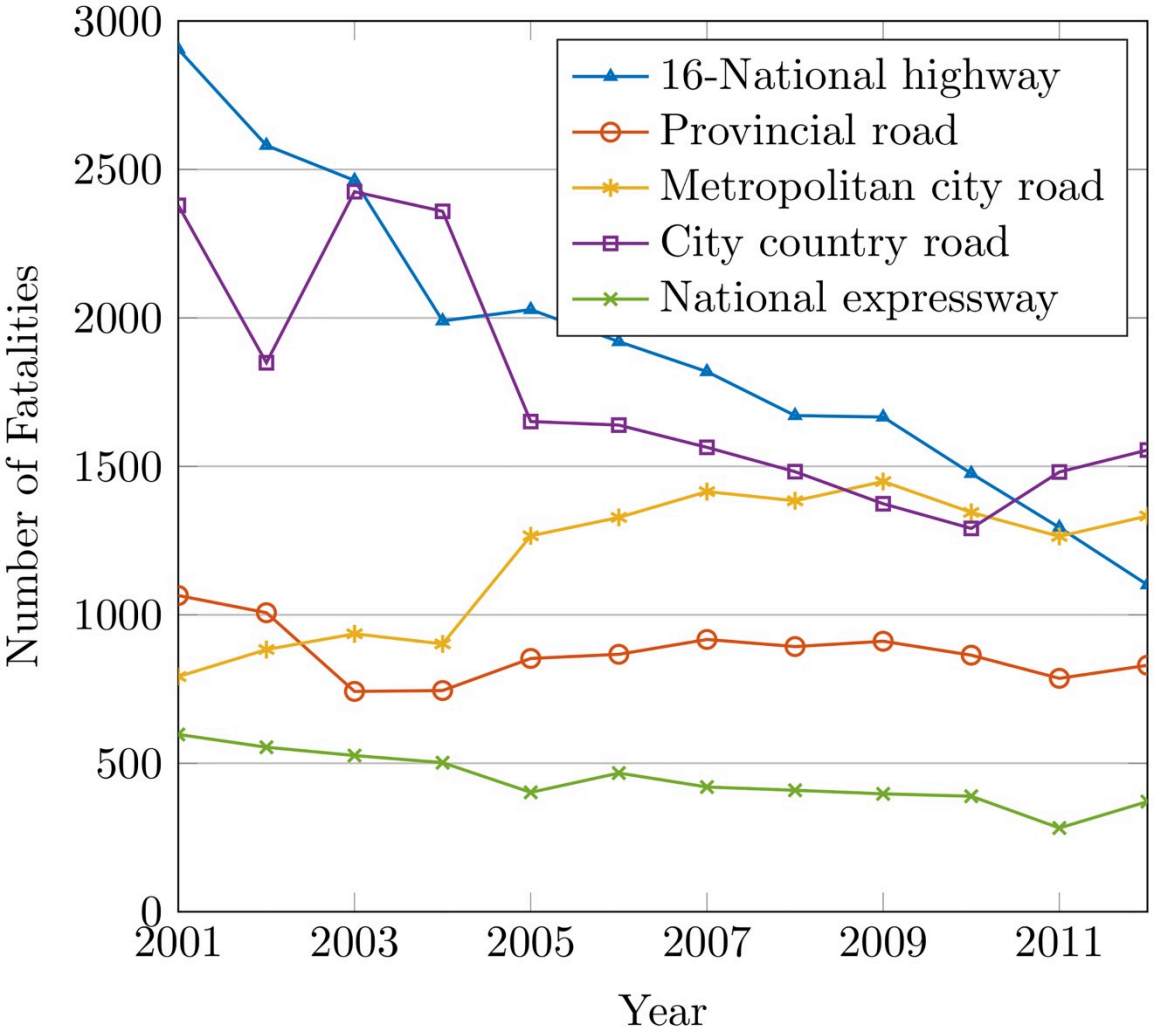
The accidents by type of road.

Fig 9 shows that the roads in the city-country and metropolitan-city have the highest fatality rate. The provincial-road and national expressway have comparatively lower accident rates from 2011 and 2012. The accident rate for metropolitan city road is found pernicious when approaching the city-country road for 2011 and 2012. The city-country road has the highest accident rate with an alarming increase at the time. As shown in Table 4, 43% of the accidents occur on the metropolitan-city road from 2009 to 2012. The analysis shows that road type has very little impact on accidents. The value of *R*^2^ is 0.00299 which proves that road type causes road crashes at minimal. The P-value is 0.93 at 95% confidence which is insignificant. A variety of road factors like allowable speed, road structure, junctions, sections lengths, etc. can influence accidents on roads which involves the demanding analysis of these factors to better understand the relationship between road type and accident rates.

**Table 4:** The accidents by type of road from 2009 to 2012.

Year	Accidents				
	Highway	Pro. road	Metro road	City road	Exp. way
2009	36,056	20,308	103,145	65,467	3,748
2010	32,479	19,907	98,517	64,616	3,924
2011	28,093	18,241	97,066	69,003	3,800
2012	19,635	19,844	95,093	75,180	3,550
**Percentage of total accidents**					
2009	15.54	8.75	44.46	28.22	1.62
2010	14.32	8.77	43.42	28.48	1.73
2011	12.67	8.23	43.78	31.12	1.17
2012	8.78	8.87	42.52	33.61	1.59
Change	-6.76	0.12	-1.94	5.39	-0.03
*R*^2^	0.003	P-value	0.093	–	–

#### Driver’s age and road accidents

Intermittent observations can be found in [45–47], where the risk of the accident is associated with the age factor. The common notion is that young people are more susceptible to inspirational pressure and thrill, poor decision making in emergency conditions, drunk driving, over speed and other traffic violations compared to mature drivers. On the other hand, elderly people are prone to fatigue and unable to do proper estimation while braking or overtaking vehicles. Moreover, the lack of control by elderly drivers in suddenly altered scenarios may lead to disastrous outcomes [48, 49].

However, [50] has a different conclusion contrary to prevalent comprehension that the increased age is more susceptible to the risk of crashes. It claims that if the comparison is done on the basis of accidents-per-km traveled, then younger and elder age groups both are endangered by the same proportion of risk. So, the accident data is divided into 8 different groups based on the age of the driver to better understand the influence of age on road accidents in South Korea. Table 5 categorizes the accidents and calculates their ratio by the age group of the drivers. The percentage of fatalities is calculated for each age group from 2009 to 2012, which shows that people of age from 51 to 60 years (in Group 6) and over 65 years (in Group 8) are liable for 49.73% of total fatalities on average. There is an average increase of 2.94% and 3.29% in fatality rate for Groups 6 and 8, respectively.

**Table 5:** The categorization of accidents based on age group of the driver from 2009 to 2012.

Group	Age	Percentage of fatalities				*Change %
		2009	2010	2011	2012	
1	Under 14	2.64	2.91	1.93	1.87	-0.76
2	15-20	4.61	5.23	5.70	4.01	-0.60
3	21-30	11.80	11.04	10.14	9.12	-2.68
4	31-40	10.69	10.55	10.15	10.00	-0.69
5	41-50	16.41	16.44	15.36	15.19	-1.22
6	51-60	16.24	16.11	17.19	19.18	2.94
7	61-65	6.32	5.87	6.56	6.06	-0.26
8	Over 65	31.28	31.83	32.97	34.57	3.29

* Negative (-) sign indicates the decrease in the accident percentage. The given change is since 2009.

**Fig 10:**
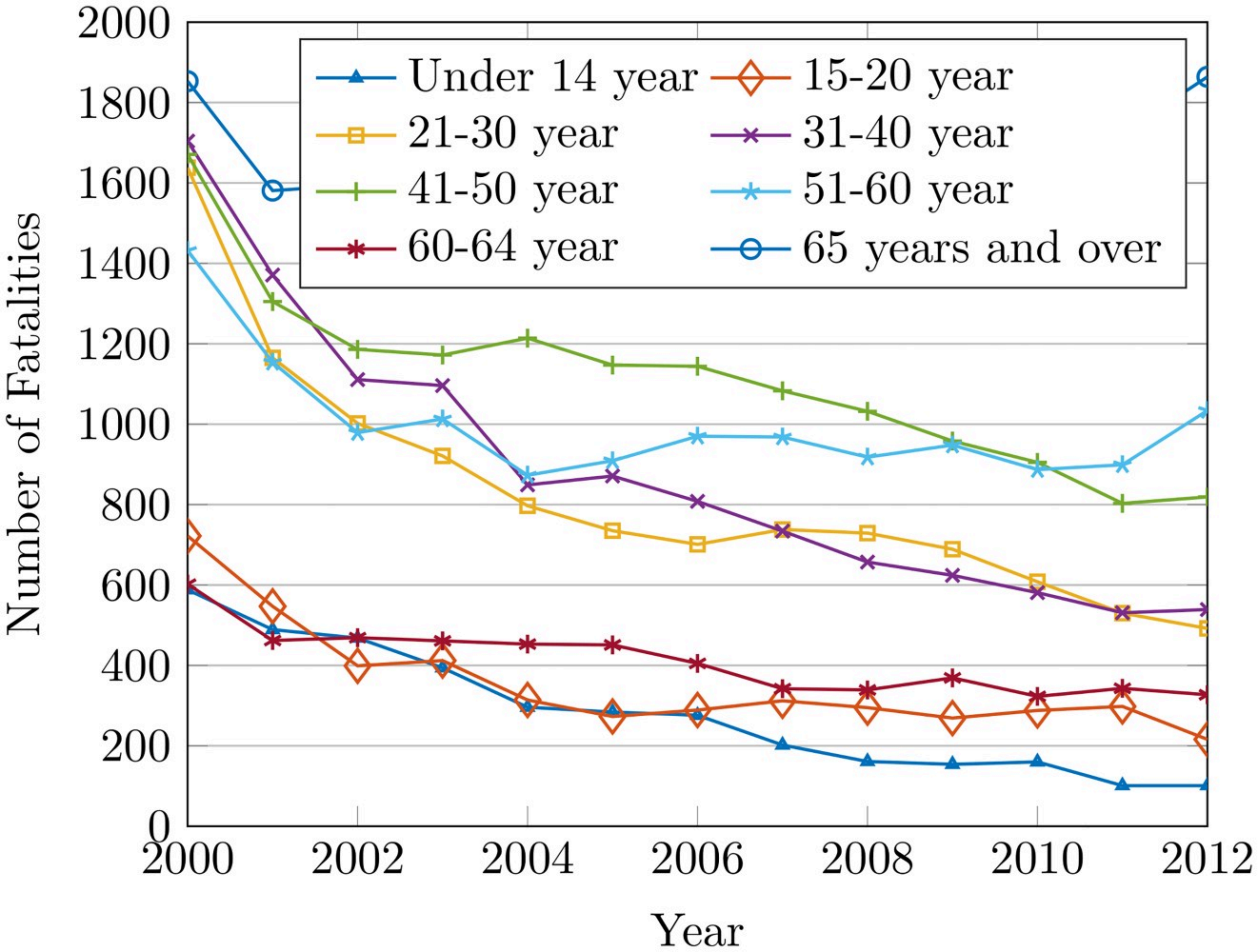
The fatalities by age group of the drivers from 2000 to 2012.

**Fig 11:**
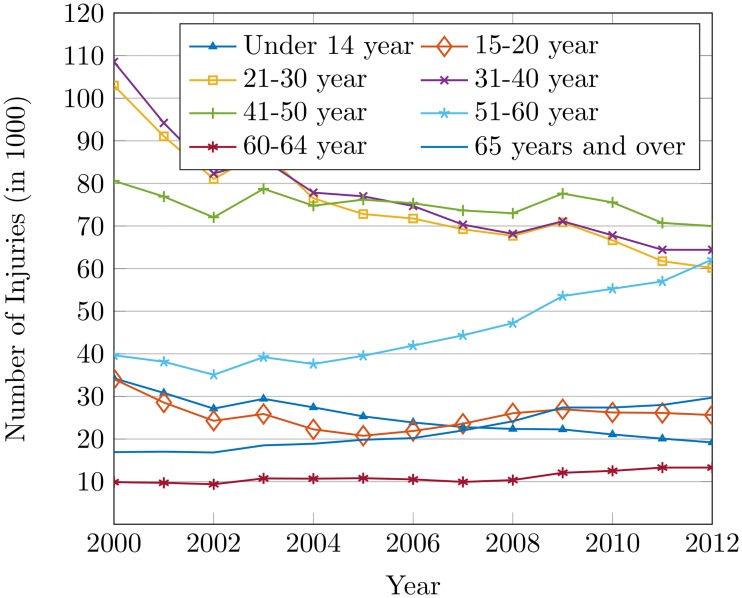
The injuries by age group of the drivers from 2000 to 2012.

Figs 10 and 11 show that the effect of age is not only the cause of the increase in the number of accidents but it raises the number of injuries as well. Drivers with age between 51 to 60 years are at an elevated risk of accidents, which might be attributed to a variety of factors. Elderly aged drivers suffer from medical conditions like arthritis, hypertension, heart disease, stroke, etc. that could influence the higher rate of accidents [51]. The inability of elderly drivers to make proper estimations of speed, the closeness of vehicles, turns, and vehicle altogether contributes to accidents as well.

The nervous actions like braking too quickly, making wrong turns, lack of vehicle control during suddenly altered situations also lead to severe crashes. Moreover, aging also affects the visual system that results in missing the approaching vehicle, pedestrians, narrowly avoiding collisions, misread signs and missing traffic signals at intersections and so lead to ruinous road accidents [52]. Visual impairment is one of the leading cause of accidents in elder age drivers [53]. Similarly, light conditions also play their part in accidents in prolonged driving at night time, which restricts the ability of elderly drivers to act vigilantly [54]. Nevertheless, the absolute correlation cannot be established fully in the absence of other relevant data like distance traveled by each age group, traveling environment (risky travel, congestion), health conditions of the drivers, road type, and so on.

#### Role of day and night in road accidents

Night makes one-third of total day time on average. However, 40% of the total fatalities and injuries occur during the night time [55]. Such statistics indicate potential danger that drivers face when driving during the night time. Furthermore, the different time during the night has a different associated risk of death and injury [56]. Driving during 2:00 am to 5:00 am includes 5.6 times increased risk of traffic accidents.

**Fig 12:**
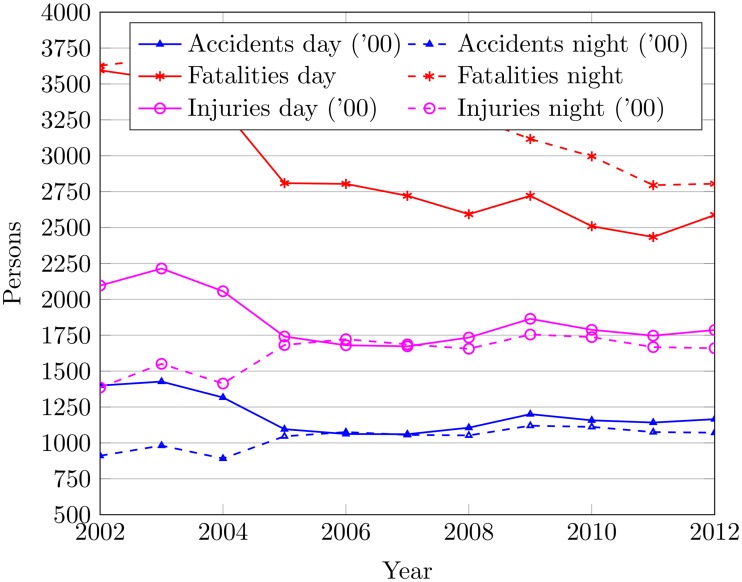
The accidents by day and night 2000 to 2012.

The researchers have various reasons for accidents rates during night time. The most general reason is the low visibility conditions and sleepiness [57]. However, another finding is that accidents during night time are more related to the use of roads at night rather than the darkness at night. So, a breakdown of accident data of South Korea is indispensable to understand the impact of night time on the accidents rate. Accidents data reveal that South Korea has a higher accident rate during day time than at night as shown in Fig 12. The most probable reason is the public preference to avoid traveling during the night time. Even so, an increase in accidents during the night is observed from 2008 to 2012. Similarly, injuries during day time are comparatively more than at night. However, the injury rates for day and night are almost equal for recent years. The number of fatalities for the day is less than that of the night time. This exemplifies that accidents during the night are severe and merciless than the day time accidents.

Table 6 shows the total accidents during day and night time to find out the number and severity of accidents from 2002 to 2012. The ratio of the night to day time accidents is increased by 7.13% on average since 2002. Similarly, an increase of 8.35% in fatality rate and 8.48% in injury rate is observed in night time accidents. Moreover, nighttime accidents are increased by 17.64% since 2002. 46.54% of the total accidents occur during the night time on average. The injury and fatality rate for nighttime accidents is observed as 46.85% and 53.38%, respectively.

**Table 6:** The accidents and fatalities during day and night from 2002 to 2012.

Year	Accidents		Accidents %		Fatality %		Injury %	
	Day	Night	Day	Night	Day	Night	Day	Night
2002	139,963	91,063	60.58	39.42	49.78	50.22	60.20	39.80
2003	142,700	98,132	59.25	40.75	48.99	51.01	58.82	41.18
2004	131,634	89,121	59.63	40.37	50.71	49.29	59.26	40.74
2005	109,560	104,611	51.16	48.84	44.06	55.94	50.86	49.14
2006	106,215	107,530	49.69	50.31	44.32	55.68	49.38	50.62
2007	106,055	105,607	50.11	49.89	44.13	55.87	49.80	50.20
2008	110,604	105,218	51.25	48.75	44.17	55.83	51.15	48.85
2009	120,013	111,977	51.73	48.27	46.61	53.39	51.51	48.49
2010	115,719	111,159	51.00	49.00	45.58	54.42	50.72	49.28
2011	114,181	107,530	51.50	48.50	46.55	53.45	51.17	48.83
2012	116,529	107,127	52.10	47.90	47.98	52.02	51.85	48.15
Average	119,379	103,552	53.46	46.54	46.62	53.38	53.15	46.85
Change	-16.74	16.74	-7.13	7.13	-8.35	8.35	-8.48	8.48

**Fig 13:**
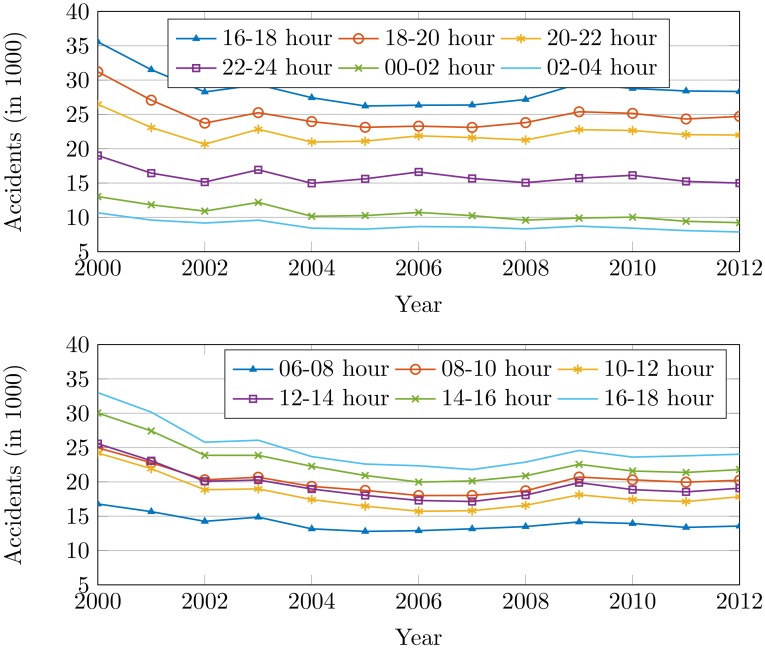
The accidents by the time of the day from 2000 to 2012.

The highest accident rate is from 18:00 to 20:00 hours of day time as shown in Fig 13. 16:00 to 18:00 and 20:00 to 22:00 hours are found to be very dangerous for traveling. The said hours of the day have increased traffic volume which leads to inflating the number of accidents.

#### Alcohol intoxication and road accidents

The percentage of alcohol in the blood of an individual according to his/her weight is often referred to as Blood Alcohol Concentration (BAC), which has an inverse impact on the performance of human beings. The human body ultimately deteriorates with the increase in BAC level. We can find various studies such as in [58, 59] which proves an established fact that drunk driving is a major factor for accidents with severe injuries.

[60] articulates that increased use of alcohol during or before driving is always a cause of increased accidents and casualty rate. Alcohol consumption varies for different age levels. Different countries have defined various BAC levels to mitigate the havoc caused by alcohol drinking. South Korea has also restricted the maximum allowable BAC level to 0.05% similar to many European and Asian countries. Drunk driving has different accident rates for male and female drivers [61]. In this regard, the use of alcohol has a different influence on drivers based on their gender, age and locality. So, an analysis of accidents is desirable to understand the effect of alcohol on drivers in Korea.

**Fig 14:**
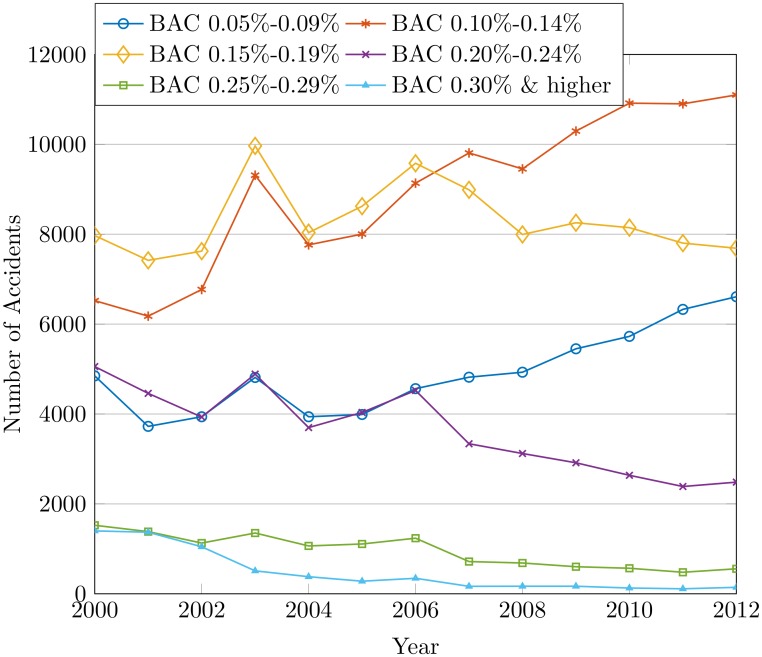
The accidents by alcohol concentration in blood of drivers from 2000 to 2012.

The drunk drivers are divided into different categories based on the BAC level. The percentage of fatalities and injuries in each group due to alcohol use is shown in Table 7. The underlying objective is to find the probability of fatalities and injuries due to drunk driving. It is observed that the chances of drivers getting killed in an accident are 24% when the BAC level is 0.30% or more. However, the group with a BAC level of 0.10% to 0.19% is more exposed to injuries than other groups. Fig 14 shows the trend of accidents, injuries, and fatalities happened due to alcohol from 2008 to 2012. This reveals that the number of accidents for the BAC levels of 0.05%-0.09% and 0.15%-0.19% has been tremendously high.

**Table 7:** The injuries/fatalities by alcohol concentration in blood of drivers from 2009 to 2012.

BAC	Percentage of fatalities to accidents				Avg.
	2009	2010	2011	2012	
0.05%-0.09%	3.27	2.67	2.75	4.42	3.35
0.10%-0.14%	2.34	2.08	1.70	1.89	2.14
0.15%-0.19%	3.27	2.66	2.32	1.98	8.58
0.20%-0.24%	4.60	4.89	5.15	3.82	4.85
0.25%-0.29%	9.63	7.92	9.60	7.91	9.18
0.30%-0.34%	11.11	6.73	17.07	9.82	11.28
0.35% & over	0.0	4.17	20.39	21.88	12.57
**Percentage of injuries to accidents**					
0.05%-0.09%	179.73	178.99	176.26	179.74	178.58
0.10%-0.14%	180.85	180.40	182.04	180.60	181.06
0.15%-0.19%	181.82	181.68	178.03	181.05	181.00
0.20%-0.24%	176.78	172.80	179.30	178.70	177.79
0.25%-0.29%	173.75	168.13	174.74	169.42	170.82
0.30%-0.34%	151.39	189.42	157.32	184.82	167.98
0.35% & over	137.50	154.17	196.55	156.25	160.51

#### Accidents by type of vehicles

Numerous factors take part in defining the severity and frequency of road accidents. However, the type of vehicle involved is one of the leading factors. According to the police statistics reported in South Korea, 151,190 accidents in total are caused by the car drivers for 2012. Only car accidents constitute 68% of the total accidents in 2012. The vehicle causing the accidents from 2009 to 2012 are categorized in Table 8. The obvious and leading reason in this regards is the increased number of cars in South Korea since passenger cars contribute two-third of the total accidents.

**Table 8:** Total accidents by type of vehicle from 2009 to 2012.

	Passenger car	Truck	Bus	Motorcycle	Bicycle	Special car
2009	143,984	30,366	15,916	10,629	2,130	1,125
Total %	68.15	13.50	7.29	4.97	1.14	0.47
2010	158,101	30,281	16,670	10,950	2,663	1,035
Total %	67.61	13.35	7.35	4.83	1.17	0.46
2011	149,864	29,143	16,236	10,170	2,883	980
Total %	67.59	13.14	7.32	4.59	1.30	0.44
2012	151,191	29,011	16,408	10,415	3,547	999
Total %	67.60	12.97	7.34	4.66	1.59	0.45

#### Accidents by violation type of driving vehicle

There exist two major characteristics of the drivers that are errors and violations, which seems responsible for accidents. Errors are flaws in human judgments and acts according to the situation while violations are deliberate actions to bypass the system. The violations of traffic laws often lead to aggravating the number of accidents. Hence, categorization of road accidents by nature of violation may lead to better help policymakers devise systems to restrict or reduce violations. Table 9 contains 5 severe and 5 minor violations that are in part responsible for road accidents in South Korea. Infringement of safe driving is the leading violation by drivers who face accidents. Seemingly, safety factors like the use of a seat belt, eating while driving, etc. may be considered as trivial even they cause the majority of accidents in Korea.

**Table 9:** The types of violations.

Violation	Code
***Severe***	
Infringement of safe driving	S-ISF
Violation of traffic signal	S-VTS
Too close to vehicle ahead	S-CTV
Improper at intersections	S-IAI
Intrusion to median strip	S-IMS
***Minor***	
Speed limit violation	M-SPL
Improper overtaking	M-IMO
Pedestrian carelessness	M-PDC
Defect in vehicle maintenance	M-DVM
Driving while fatigued	M-DWF

In South Korea, 56.064% of the accidents are led by safety driving violations as shown in Table 10. In addition, a gradual increase in infringement of safe driving has been observed since 2012. Other violations included in the severe group are the violation of traffic signal, driving too close to vehicles going ahead, driving improperly at intersections and intruding the median strip. Many researchers have investigated the role of driving speed in road crashes and proven that higher driving speed is associated with higher chances and severity of accidents [62, 63]. As shown in Table 11, the violation speed limit makes only 0.18% of the total accidents.

**Table 10:** The accidents caused by severe violations.

	S-ISF	S-VTS	S-CTV	S-IAI	S-IMS
2009	126,340	27,582	24,554	17,145	14,327
Total %	54.47	11.89	10.59	7.39	6.18
2010	125,082	25,963	23,126	16,206	14,071
Total %	55.13	11.44	10.19	7.14	6.20
2011	123,744	24,504	22,315	15,172	12,931
Total %	55.85	11.05	10.07	6.84	5.83
2012	125,391	25,307	22,275	14,721	13,018
Total %	56.06	11.32	9.96	6.58	5.82

**Table 11:** The accidents caused by minor violations.

	M-SPL	M-IMO	M-PDC	M-DVM	M-DWF
2009	422	94	12	16	2
% of total	0.182	0.041	0.005	0.007	0.001
2010	403	74	14	8	5
% of total	0.178	0.033	0.006	0.004	0.002
2011	403	82	8	11	0
% of total	0.182	0.037	0.004	0.005	0
2012	377	89	9	14	5
% of total	0.169	0.040	0.004	0.006	0.002

#### Driving experience of drivers involved in accidents

One common perception is that higher driving experience decreases the number of accidents since it makes drivers better judge perilous situations [64, 65]. Experience makes drivers avoid risky scenarios and reduce violations as well. The accidents are categorized according to the driving experience of the driver in Table 12 that shows the contradictory results to the given notion. In South Korea, 42% of accidents are caused by drivers on average whose driving experience is 15 years or more. Accidents rate is decreased while the driving experience is increased from 1 to 5 years. However, after 5 years of driving experience accident rate is increased quickly. It constitutes three fourth of the total accidents if we consider the driving experience of 5 years or more. Worse still, the ratio of accidents by drivers with 15 years or more in driving experience is elevated by 4.24% since 2009. The most persuasive reason is the elder age of the drivers with 15 years or more driving experience. A separate analysis considering driving experience with the age of the driver may help understand the leading cause of increased accidents for the experienced drivers.

**Table 12:** The percentage of accidents under the driving experience of the first party.

Experience	2009	Total %	2010	Total %	2011	Total %	2012	Total %
< 1 yr	11,332	4.89	10,890	4.80	10,245	4.62	10,809	4.83
1-2 yrs	8536	3.68	8346	3.68	7878	3.55	7792	3.48
2-3 yrs	7161	3.09	7810	3.44	7223	3.26	7162	3.20
3-4 yrs	7892	3.40	6849	3.02	7031	3.17	6783	3.03
4-5 yrs	7561	3.26	7562	3.33	6376	2.88	7087	3.17
5-10 yrs	44724	19.28	41032	18.09	36726	16.57	33554	15.00
10-15 yrs	34817	15.01	33491	14.76	31967	14.42	35162	15.72
15 & over	91161	39.30	92840	40.92	96323	43.45	97376	43.54

#### Vehicles traveled kilometers and accidents

The analysis of the total number of registered vehicles may not be enough to determine the role of vehicles on accidents and fatalities. Therefore, vehicles’ total traveled kilometers for specific areas are analyzed to find out their impact on the accident fatalities. For this purpose, three cities are selected each with the highest and lowest number of accidents. Table 13 shows the data for total vehicle travel and total deaths for selected cities. Analysis reveals that traveled km has little impact on the number of fatalities in Korea. Cities with a lower number of registered vehicles and traveled km tend to have a higher number of deaths. On the contrary, cities like Seoul, Busan, and Daegu have a higher number of deaths in general. However, the deaths/1 billion km is less than those of Daejon, Ulsan and Jeju when compared on the basis of km traveled.

**Table 13:** Vehicles’ traveled kilometers and number of accidents.

Division	Vehicle total km traveled			Total deaths			Deaths/1 billion km		
	2012	2013	2014	2012	2013	2014	2012	2013	2014
Seoul	42,816,820,954	40,925,305,723	41,912,328,228	419	371	399	7.80	7.00	7.40
Busan	18,194,288,769	17,762,287,074	18,086,593,635	223	207	165	10.20	9.60	7.50
Daegu	14,824,319,979	14,833,897,650	15,345,327,933	187	157	173	11.90	9.50	10.10
Daejon	8,511,744,532	8,387,804,048	8,705,544,423	121	87	97	12.10	10.40	9.40
Ulsan	6,542,462,280	6,552,603,994	6,833,347,368	101	119	102	14.30	15.50	11.60
Jeju	4,375,973,885	4,908,236,632	6,031,174,664	92	107	92	18.30	18.80	13.80

#### Impact of population density on accidents

The population and registered vehicles information can be helpful to interpret the accidents in Korea as population density can be related to accidents frequency. Research in [66] indicates that population density has a strong impact on the accidents frequency. This points out that lower population density areas have higher casualties while areas with higher population density experience lower casualties. Similarly, [67] draw a similar conclusion where the decreasing population density is associated with an increased number of accidents. The population density and accidents data for Korea is gathered to analyze their correlation. Table 14 shows this data for different cities of Korea.

**Table 14:** Population density and number of accidents.

Division	Population density/km^2^			Accidents		
	2005	2010	2015	2005	2010	2015
Seoul	16,221.0	16,188.9	16,364.0	38,610.0	41,662.0	41,665.0
Busan	4,609.4	4,452.3	4,479.9	11,873.0	13,847.0	12,757.0
Daegu	2,786.5	2,767.4	2,791.0	12,252.0	14,600.0	14,228.0
Incheon	2,546.3	2,587.5	2,755.5	11,709.0	10,305.0	8,727.0
Gwangju	2,827.5	2,945.6	2,998.8	7,797.0	8,894.0	7,864.0
Daejeon	2,673.0	2,781.2	2,852.3	5,399.0	5,870.0	6,901.0
Ulsan	992.5	1,022.3	1,099.6	4,359.0	5,067.0	5,368.0
Gyeonggi-do	1,028.1	1,119.3	1,226.4	43,595.0	43,963.0	52,954.0
Gangwon-do	88.2	88.2	90.2	8,682.0	9,026.0	8,912.0
Chungcheongbuk-do	196.5	203.4	214.6	7,673.0	8,571.0	9,335.0
Chungcheongnam-do	219.7	235.0	256.6	8,646.0	9,282.0	9,421.0
Jeollabuk-do	221.5	220.3	227.4	9,823.0	10,453.0	8,873.0
Jeollanam-do	150.7	142.2	146.1	10,472.0	10,949.0	10,420.0
Gyeongsangbuk-do	137.1	136.6	140.8	16,772.0	16,498.0	15,752.0
Gyeongsangnam-do	290.5	300.0	316.4	13,343.0	14,274.0	13,677.0
Jeju-do	287.8	287.7	327.5	3,166.0	3,617.0	4,645.0

The regression analysis results indicate that population density has a strong impact of accidents frequency with *R*^2^ value as 0.31 and a significant *P* value as 0.28 at 95% confidence. The results are in conformity with previous research as lower population density is involved with a higher number of accidents while the higher population density is coupled with a reduced number of accidents.

### Multiple factor analysis

This section evaluates how multiple factors cause accidents when they are combined. However, data analysis becomes complicated when multiple variables are considered collectively. Hence, only the accidents that involve fatalities are considered from 2009 to 2012.

#### Driver experience, accident time and gender

The regression analysis is performed to investigate the impact of three variables on fatalities. Fatalities are considered with respect to the experience of the male and female drivers in addition to the occurrence time of the accident. The results are summarized in Table 15, which shows that gender and driving experience plays a pivotal role while the accident time has a little impact on accidents. Higher weight is associated with gender and then driving experience. The value of *R*^2^ is 0.54 which indicates that 54% accidents can be attributed considering the given three factors together with a significant *P* value. Statistics show that male drivers have higher tendencies to be a victim of accidents as their driving experience increases. The fatalities in nighttime accidents with senior drivers are higher than those in the day time. However, this difference in deaths during day and night time is more prevalent for the male drivers with driving experience from 1 to 10 years. The fatalities of drivers with an experience of more than 10 years are similar during the day and night time. Contrary to male drivers, female drivers are more susceptible to fatality during the day time. Moreover, higher fatalities are associated with female drivers as long as their driving experience increases from 3 to 15 years. The fatality rate is less when the experience is higher than 15 years. Male drivers are attributed to 36.29% and 53.01% of total fatalities during the day and nighttime, respectively. On the other hand, female drivers respectively have 5.64% and 5.06% fatalities in day and night.

**Table 15:** The fatalities with driving experience, gender, day and night from 2009 to 2012.

**Year**	**2009**					**2010**				
**Time**		**Day**		**Night**			**Day**		**Night**	
**Driving Exp**.	**Deaths**	**M**	**F**	**M**	**F**	**Deaths**	**M**	**F**	**M**	**F**
1 year	221	67	11	130	13	245	84	10	139	12
2	218	89	8	107	14	230	68	17	128	17
3	185	58	11	105	11	161	55	5	95	6
4	168	63	10	87	8	157	52	14	82	9
5	208	81	12	106	9	194	66	7	116	5
10	1138	419	64	578	77	966	320	81	509	56
15	811	328	70	358	55	802	300	66	381	55
> 15	2304	1082	71	1080	71	2243	1030	86	1052	75
**Year**	**2011**					**2012**				
**Time**		**Day**		**Night**			**Day**		**Night**	
**Driving Exp**.	**Deaths**	**M**	**F**	**M**	**F**	**Deaths**	**M**	**F**	**M**	**F**
1 year	206	65	12	119	10	237	77	19	124	17
2	177	57	12	98	10	181	66	12	98	5
3	173	50	12	104	7	175	53	8	107	7
4	159	46	5	97	11	149	51	5	89	4
5	164	64	6	84	10	163	60	4	89	10
10	904	324	78	452	50	777	297	47	406	27
15	683	259	50	339	35	799	315	55	375	54
> 15	2277	1068	95	1043	71	2442	1157	117	1087	81

#### Driver age, violation type, and type of accidents

The age of the drivers, violation type, accident type and the number of fatalities for each type are analyzed from 2009 to 2012. Fatalities are categorized with respect to 5 age groups including ≤ 20, 21-30, 31-40, 41-50, 51-60 and 61 & above. Five major violations and four accident types including *‘car vs human’*, *‘car vs car’*, *‘single vehicle’* and *‘railroad crossing’* are shown in Table 9. Therein, *R*^2^ value of regression is observed as 0.35 with significant *P* value. Higher weight is associated with violation type followed by the age. The analysis reveals that 29.15% of total fatalities of the drivers with the age of 41-60 have resulted from safety violations. So, 18.14% of accidents between the car and human and that of 18.33% between the car and car happened. Drivers with age ≤20 make fewer violations and fatalities. Similarly, drivers with age of 21-40 make safety violations which lead to the accidents of the car with the car and single vehicles.

## Key results and insights

Traffic volume and rain are found to be the leading factors that define the frequency and severity of the accidents. The increased rain is hazardous for traffic and so leads to accidents. The road length and number of vehicles constitute 85% of the total accidents. The road is extended with a moderate rate of 1.47% against the quick increase of 4.14% in the number of vehicles annually. The number of vehicles has grown by 69% while the road is extended by 20% only since 2000. This huge gap has created increased traffic volume and congestion. The majority of the registered vehicles are passenger cars. Folks preference for cars over public transportation is another cause of accidents. Only passenger cars are involved in almost two-thirds of road accidents such that 151,190 car accidents in 2012 are 68% of the total accidents. The cars population is highly likely to be controlled and public transportation should be encouraged at least for intra-city traveling to control the high number of accidents. The over speed is a violation that only makes 0.18% of the total accidents and severe injuries. On the other hand, infringement of safe driving is the leading violation by drivers and 56.064% of total accidents happen due to safety driving violations in 2012. Male drivers are responsible for 74% of total accidents and they are attributed to 3.17 times higher accidents than that of female drivers.

The experienced drivers are considered very precise in driving habits and following traffic rules and so are less likely to cause accidents. However, 42% of accidents are caused by drivers whose driving experience is 15 years or more in South Korea. The accident rate decreases while the driving experience is between 1 to 5 years. However, after 5 years of driving experience accident rate is increased. Almost three fourth of total accidents are attributed to drivers with driving experience of 5 years or more. This may be due to the high age of the driver, slow reflex actions, violations, drunk driving, etc. The driving experience alone may not be a good indicator to indicate the root causes of accidents. Thus, multiple variables are considered in the analysis as well. The analysis uncovers that the driving experience along with the gender of the driver and the time of the accidents in terms of day and night time helps to further scrutinize the underlying causes. Results show that male drivers with higher driving experience are more vulnerable to fatal accidents during the night time than those of the female drivers. This difference in deaths during day and night time with male drivers is more evident with drivers having experience from 1 to 10 years. On the other hand, drivers with experience of 10 years or more have equal chances of fatal accidents during day and night. The multivariate analysis shows that higher weights are associated first with the violation type followed by the driver age group. The driver with ages from 41 to 60 commit safety violations and so leading to 29.15% fatal accidents involving car vs human and car vs car. Drivers with age ≤ 20 are among the least violators while the driver with age from 21 to 40 years commit safety violations and lead to car and car and single vehicles accidents.

The light conditions also play their part in accidents. The accidents during the day are higher than at night in South Korea. However, nighttime accidents are so perilous and severe that death at night time is 8.35% higher than that of daytime. Night makes an average of 46.54% accidents. However, the fatality rate is 53.38% for night accidents. The night accidents have been elevated by 7.13% since 2002. The drunk driving is another reason for an increase in accident and fatality rate. Analysis reveals that there are 24% chances of getting killed in an accident when driving with a BAC level of 0.30% or more. However, most of the injuries are caused when the BAC level is between 0.10% to 0.19%. The analysis showed both compatible and contradictory results with the previous research. Some of the results may be pertaining to the cultural, social and demographic variations of South Korea from other countries. This study’s findings are believed to be helpful for authorities to understand the factors responsible for road accidents and devise better policies to mitigate their effects.

### Propositions

More roads are needed to accommodate the increasing demand for traffic day by day. However, the Government can consider three very effective strategies otherwise: 1) Restriction of the mobile phone use during driving; 2) Use of speed cameras, and 3) Variant Message Systems (VMS). The use of the mobile phone during driving has an associated risk of 120% compared with driving without using a mobile phone [68]. The deployment of speed cameras can make a significant decrease of 18% in accidents [69]. The VMS undoubtedly reduces accidents, most profoundly when used for accidents and fog warning messages. The scientific community is eager to play its vital part to tackle other factors including light conditions, altered weather and assisting the elder aged drivers to mitigate the problems caused by visual impairment with the help of Advanced Driver Assistance Systems (ADAS). In the UK, the Electronic Stability Control (ESC) is proved to reduce accidents by 3%. In Germany, Sweden, and USA it is noticed as 45%, 22%, and 41%, respectively [70, 71]. This analysis urges the need to make use of the sensors which can work in altered light and weather conditions. Studies [72, 73], [74] prove that use of Night Vision Enhancement Systems (NVES) like the use of LIDAR and Forward Looking InfraRed (FLIR) thermal cameras can increase visibility up to 250 meters to 300 meters which are normally 30 meters to 50 meters for dark objects when older drivers are considered. The NVES can potentially reduce the number of crash fatalities by 20 to 25%. Since cars are responsible for 68% of accidents, so, equipping cars with such sensors will help drivers driving safely and alleviating the number of accidents in Korea. In the same fashion, advanced investigative technologies using traffic and accident simulators can also help understand the root causes of road accidents [75]. For this purpose, real-world accidents data can be modeled in the simulated environment to study the cause and effect relationship of the different accident causing factors.

## Conclusions

In this paper, various leading factors are analyzed which may cause road accidents in South Korea. The accident data is collected from various reliable sources to carry out the multivariate regression analysis. Results demonstrate that traffic volume and infringement of safety violations are the ultimate causes of road accidents in South Korea. Therein, the widespread growth of vehicles affects the traffic volume which leads to a higher number of accidents. The analysis reveals that 68% of total accidents are by passenger cars only. The probability of an accident is higher with the experienced drivers when the driving experience is 10 years or more. Male drivers have a 3.17 times higher accident rate than that of female drivers. 36.29% and 53.01% of total fatal accidents during the day and night time are respectively caused by male drivers. The age of the drivers, traffic volume and rain are critical factors for elevated road accidents. The analysis of collision type may also help to uncover accident causes, their associated risk factors and their relationship to the severity of the accident. The Government needs to revisit traffic regulations in a fast developing country like South Korea along with the lines of this study so that urban roads can be made safer to use and travel for all.
